# The Re-emergence of Human Metapneumovirus: Virus Classification, Characteristics, Mechanisms of Infection, Clinical Features, Diagnosis, Epidemiology, Prevention, and Treatment

**DOI:** 10.7759/cureus.85259

**Published:** 2025-06-02

**Authors:** Nethra Chittiprol, Venkataramana Kandi, Venkata Bharat Kumar Pinnelli, Tarun Kumar Suvvari, Naveen Madamsetti, Jayashankar CA, Sai Teja Challa

**Affiliations:** 1 Health Science, Cypress Bay High School, Weston, USA; 2 Clinical Microbiology, Prathima Institute of Medical Sciences, Karimnagar, IND; 3 Biochemistry, Vydehi Institute of Medical Sciences and Research Centre, Bengaluru, IND; 4 General Medicine, Rangaraya Medical College, Kakinada, IND; 5 Research, Squad Medicine and Research (SMR), Amadalavalasa, IND; 6 Inhalation, Proveris Scientific Corporation, Hudson, USA; 7 Internal Medicine, Vydehi Institute of Medical Sciences and Research Centre, Bengaluru, IND; 8 General Medicine, Vydehi Institute of Medical Sciences and Research Centre, Bengaluru, IND

**Keywords:** coronavirus disease 2019 (covid-19), human metapneumovirus (hmpv), pandemic, re-emergence, respiratory tract infections, severe acute respiratory syndrome coronavirus (sars-cov-2)

## Abstract

The respiratory virus known as the human metapneumovirus (HMPV) was discovered for the first time in 2001 in the Netherlands. It is a ribonucleic acid (RNA) virus that belongs to the Paramyxoviridae family. It causes upper and lower respiratory tract infections (RTIs), especially in young children and the elderly. Although the majority of HMPV infections are resolved on their own, some infected infants, children, and elderly patients need to be hospitalized. Patients with underlying immunodeficiency diseases, transplant recipients, and those with other co-morbidities, such as chronic diseases, are more likely to develop complications from HMPV infections, such as pneumonia. The symptoms of HMPV infections are similar to those of other viral RTIs caused by respiratory syncytial virus (RSV), influenza viruses, and coronaviruses. Differential diagnosis and identification of the etiological agents responsible for RTIs are crucial for improved patient care. Concerns of the next pandemic have been fueled by the discovery of the novel severe acute respiratory syndrome coronavirus-2 (SARS-CoV-2), the causative agent of the coronavirus disease-2019 (COVID-19) that caused the pandemic, and the recent advent of other viral diseases like mpox. In addition, the World Health Organization (WHO) has emphasized the importance of public health readiness, as many pandemics are expected to occur. In light of this and a recent increase in HMPV cases signifying its potential re-emergence, we tried to thoroughly examine and update information on origin, transmission, pathogenicity, clinical features, laboratory diagnosis, epidemiology, prevention, and treatment of HMPV.

## Introduction and background

Infectious disease outbreaks and resurgences are impending, as seen by the recent dangers posed by viruses such as the highly pathogenic avian influenza (HPAI) virus (H5N1), Zika virus, Dengue virus, Mpox virus, and Ebola virus [1-5]. The ribonucleic acid (RNA) viruses demonstrate large variations in their genomes and show high mutation rates. The coronaviruses and the influenza viruses are the best examples of how RNA viruses can evolve and transmit from animals to humans. Many RNA viruses cause infections in animals and birds and are associated with zoonotic infections in humans. Myxoviruses are viruses that thrive in mucin secretions from respiratory epithelial cells. These viruses are divided into two types: orthomyxoviruses, which include influenza viruses, and paramyxoviruses, which include a wide range of viruses such as measles and mumps virus. Paramyxoviruses are commonly transmitted through the respiratory aerosols and frequently cause infections in children. Furthermore, several members of the Paramyxoviridaefamily have been associated with zoonotic infections like the Nipah virus and human parainfluenza virus (HPIV) types. Respiratory tract infections (RTIs) are a serious public health issue due to their high rates of morbidity and mortality, especially in children and the elderly, which lead to significant healthcare needs and financial burdens. Lower respiratory tract infections (LRTIs) impact the lungs and airways, while upper respiratory tract infections (URTIs) affect the larynx, throat, and nose.

The human metapneumovirus (HMPV) is one among the many respiratory viral etiologies causing RTIs in humans, and this virus includes respiratory syncytial virus (RSV), influenza A (IAV) and B (IBV) viruses, parainfluenza virus (PIV) types 1-3, human rhinovirus, and human coronaviruses (HCV-229E and HCV-OC43). The HMPV can cause acute RTIs in all age groups. However, children and neonates are increasingly susceptible to HMPV infection. Most children above five years develop antibodies against HMPV, revealing exposure to the virus and its presence in the environment. HMPV initially affects the upper respiratory tract like other respiratory viruses and can result in lower RTI (LRTI) affecting the lungs. More than 95% of HMPV infections occur among young children, confirming the age predilection. The disease in children commonly presents as bronchiolitis and pneumonia. Children in their first year of life are more severely affected, requiring hospitalization. Some of the predisposing factors for HMPV infection include premature birth, chronic obstructive pulmonary disease (COPD), and children with congenital heart diseases. Clinical feature-based diagnosis is difficult to suspect the exact viral etiology since other prevalent viral infections, like persons infected with RSV and Influenza viruses, also present with similar symptoms.

The HMPV infection was first reported among children in the Netherlands who were suffering from acute LRTIs. All the children from whom HMPV was isolated belonged to a less than five-year age group, with 50% of them in the six-to-12-month age group. The most common symptoms of HMPV infection are bronchitis, bronchiolitis, and difficulty breathing, attributed to airway obstruction. Scientists have observed that although the virus was newly discovered, the circulatory antibodies in most children under five years old indicate that the virus could have existed for many years. Also, human serum samples dating back to 1958 revealed HMPV-specific antibodies, highlighting its presence for years before its discovery. The genomic studies indicated that the HMPV virus has similarities with avian pneumovirus (APV) and turkey rhinotracheitis virus (TRTV) [6].

There is a recent buzz about rising respiratory illnesses on the Chinese mainland. It was observed that this was attributed to the ease of restrictions after the COVID-19 pandemic. It was noticed that there was an increase in the mortality and hospitalization rates among people infected with RTIs caused by influenza viruses, RSV, and bacterial agents like *Mycoplasma pneumoniae* and *Streptococcus pneumoniae*, compared to pre-pandemic times. This change in the dynamics of microbial etiologies in the RTIs was thought to be associated with social distancing, lockdowns, excessive use of sanitizers and disinfectants, and preventing the spread of other prevalent microbes. However, the ease of restrictions had once again created a better environment for these viruses to bounce back and cause heightened infections in the post-COVID-19 pandemic era [7-9]. Another factor that contributes to changing prevalence dynamics is viral interference mechanisms. The significance of viral interference has been validated by earlier research, which showed that RSV limits the growth of HMPV in cultures. HRV inhibits the replication of SARS-CoV-2 and IAV, RSV inhibits the replication of human rhinovirus, and IAV inhibits the replication of RSV [10].

According to data from the World Health Organization (WHO), the northern hemisphere is experiencing a surge in acute RTIs with HMPV and other seasonal viruses, such as influenza viruses and RSV. These infections are affecting North America, Europe, Asia, and Africa. The results indicate a rising tendency of HMPV infections in China and a declining incidence of RSV in North America. The WHO advocates for integrated surveillance of RTIs and their etiologies worldwide but does not recommend restrictions on people's travel and movements across nations [11]. Given the complexity of the emergence and re-emergence of RTIs caused by viral etiologies like HMPV, it is important to understand their transmission dynamics, including the pathophysiological characteristics, diagnosis, and control and preventive measures. This review comprehensively discusses the origin, transmission, pathogenicity, immunological responses, clinical features, laboratory diagnosis, epidemiology, prevention, and treatment of HMPV.

## Review

Viral classification of HMPV

**Figure 1:**
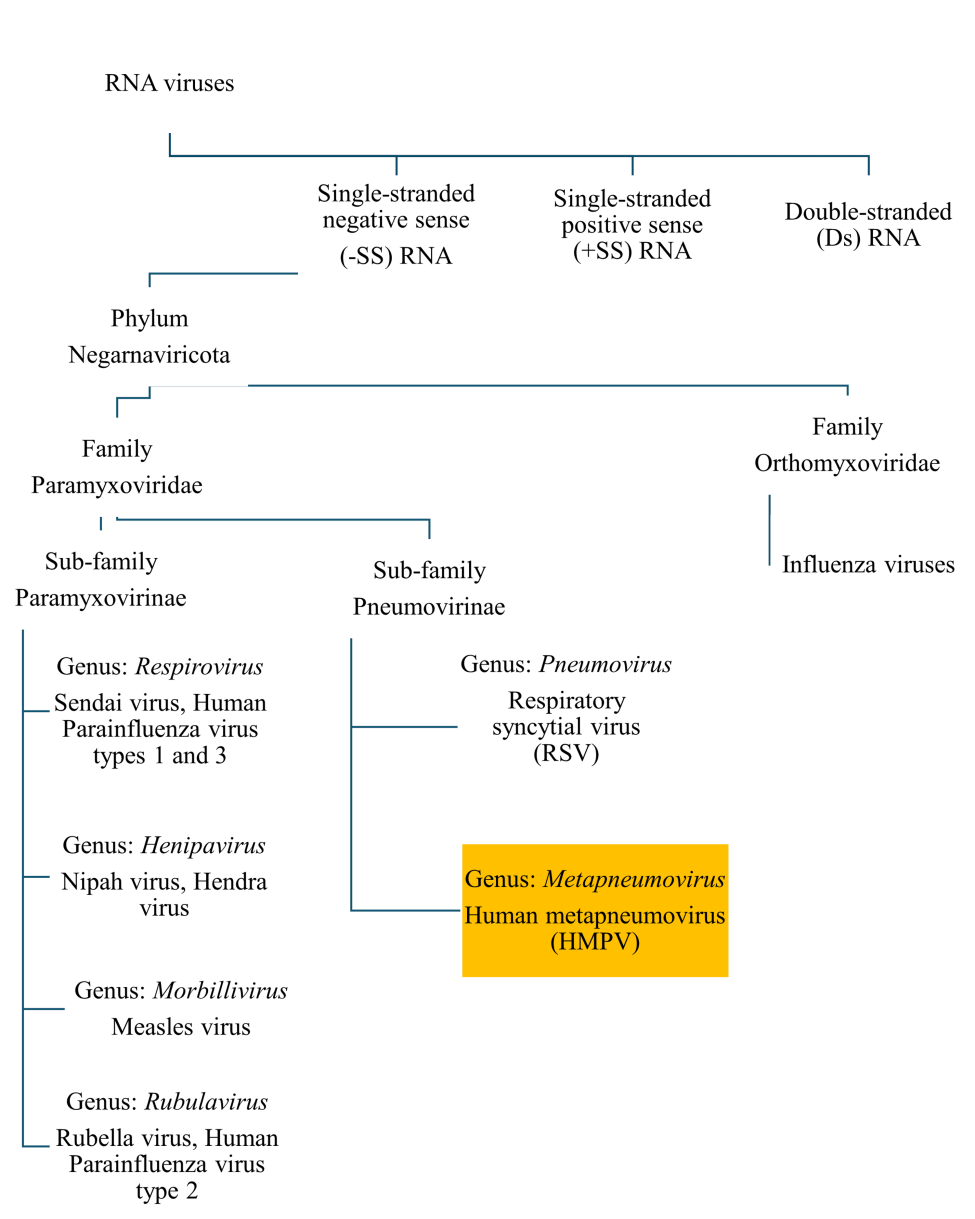
Taxonomical position of human metapneumovirus Image credit: Venkataramana Kandi RNA: ribonucleic acid

The HMPV belongs to the Paramyxoviridaefamily, which consists of single-stranded RNA viruses that are enveloped. The Paramyxoviridaefamily is classified into two subfamilies: Paramyxovirinae and Pneumovirinae. The Paramyxovirinae family is classified into four genera: *Respirovirus*, *Henipavirus*, *Morbillivirus*, and *Rubulavirus*. The HPIV types, measles virus, the mumps virus, Nipah, and Hendra viruses are some of the Paramyxovirinaeviruses causing RTIs in humans. The Pneumovirinae group contains murine orthopneumovirus and the human orthopneumovirus, consisting of RSV and the HMPV [12] (Figure 1).

Viral characteristics of HMPV

HMPV is an enveloped virus consisting of a negative-sense, non-segmented, and single-stranded RNA genome measuring approximately 13 kilobases (kb). HMPV has a diameter of 150-300 nm and is pleomorphic. HMPV occurs in two genotypes (A and B) and four subtypes (A1, A2, A3, B1, and B2). The HMPV genome codes for nine non-structural (NS) proteins including the nucleoprotein (N), phosphoprotein (P), matrix protein (M), matrix-2 proteins (M2-1 and M2-2), fusion protein (F), small hydrophobic protein (SH), glycoprotein (G) and large polymerase protein (L). HMPV genome does not code for non-structural (NS) proteins, which are found in RSV (NS1 and NS2). The structural proteins facilitate viral attachment, fusion, and penetration of host cells. The N, L, P, M2-1, and M2-2 proteins facilitate virus replication and transcription. The M protein contributes to viral assembly and budding inside the infected cells. Because of their vital roles in virus entry and cell-to-cell transmission, membrane glycoproteins such as glycoprotein G, SH, and the F protein function as molecular determinants of host specificity. The generation of neutralizing antibodies (NAbs) is stimulated by the highly immunogenic F protein. G and SH were considered to be minimally immunogenic, although they stimulate NAbs. Additionally, it was observed that the viral G protein suppresses the innate immunological responses of the host cell, hence playing a crucial role in immunomodulation. To downsize the host's immunological responses, the viral proteins also inhibit the activity of toll-like receptors (TLRs) and pattern recognition receptors (PRRs). This limits the ability to identify the virus and eliminate it from the host, which may lead to re-infections [13-15].

Transmission of HMPV

HMPV is extremely contagious, particularly among young children in close quarters like schools and childcare facilities. Like other respiratory viruses, HMPV is spread by inhalation of respiratory droplets or aerosols from coughing or sneezing, direct contact with the secretions, involving touching of HMPV-contaminated surfaces and articles before touching the face (mouth, nose and eyes), and close contact with infected individuals like sharing utensils, kissing, and other household contact. HMPV has been observed to have emerged from the avian metapneumovirus (AMPV). AMPVs have been detected in wild and domestic birds like turkeys and chickens. AMPV exists in four genotypes, such as A, B, C, and D. Molecular studies identified the AMPV-C genotype as being closely related to HMPV, suggesting it could have originated from AMPV-C. Despite the zoonotic origin of HMPV, infection in humans generally occurs through human-to-human transmission [16]. Understanding that pets are not affected by HMPV and do not pose a danger of catching the virus is vital. HMPV only infects and spreads between people. The basic reproduction number (R0) depicts the transmissibility of the virus from one person to the other person. The R0 of HMPV was found to be 1.2, meaning one infected person can transmit the infection to 1.2 persons. The R0 for seasonal influenza viral infection is 1.28, and for COVID-19, it is more than 2.0, and RSV has an R0 of 3.0 [17]. The transmissibility of HMPV appears to be similar to other seasonal viral infections.

Pathogenicity

HMPV infects respiratory epithelial cells, which triggers a pulmonary inflammatory process, releasing cytokines and mediators like thymic stromal lymphopoietin (TSLP) and interleukin (IL)-33. The infection also stimulates both Th1- and Th2-type immune responses. Secretion of IL-33 by the respiratory epithelial cells triggers an inflammatory response that exacerbates respiratory effects like asthma. TSLP and IL-33 further trigger the expression of the intercellular adhesion molecule (ICAM)-1, eosinophils, dendritic cells, IL-6, C-C motif chemokine ligand (CCL)-17, cluster of differentiation positive thymic lymphocyte (CD4+T) cells, and natural killer (NK) cells [18]. Virulence studies carried out in vitro and in laboratory animals have revealed that the clinical strains isolated from patients failed to actively grow in the laboratory cultures. The inoculation of virulent strains into lab animals induced airway dysfunction, lung damage, and death. Virulent strain infection caused a massive influx of proinflammatory cytokines, and neutrophils along with increased secretion of type I and type III interferons (IFNs), including IFN-lambda (IFN-λ) [19]. Infection with HMPV presents symptoms similar to any other RTI. HMPV majorly affects the lower respiratory tract, unlike other seasonal viral RTIs. LRTI presents as fever/feverish, cough, dyspnea, rhinorrhea, and abdominal pain. More than half of the infected patients may require oxygen supplementation. Interestingly, HMPV pneumonia was comparatively less serious than the pneumonia caused by RSV [20, 21].

HMPV infection could predispose to central nervous system complications. This was revealed in a study among children aged under 18 years. Of the 1474 cases, 63 (4.27%) were positive for HMPV and 145 (9.83%) were positive for RSV. Despite a comparatively high prevalence of RSV, infection with HMPV resulted in higher incidences of seizure (6.3%; 4) compared to RSV infection (0.7%; 1) [22]. Premature infants (140 children ≤3 years of age) have been noted to elicit a different kind of immune response to HMPV infection compared to the RSV-infected subjects. HMPV-infected premature children (<32 weeks of gestation) did not exhibit increased Th1/Th2 ratios or elevated nasal airway secretion of IFN-gamma (IFNγ), CCL5, and IL-10 [23]. HMPV-associated severe acute respiratory illness (SARI) was observed in 4.1% of patients enrolled, including 5.6% (593/10503) children and 1.7% adults (≥18 years; 119/6934). The majority of adults (84.0%) had an underlying medical condition, including HIV infection in 87/110 (79.1%) patients. The SARI incidence was higher in HIV-infected persons compared to HIV-uninfected persons in age groups 5-17 years (RR 6.0; 1.1-20.4), 18-44 years (RR 67.6; 38.0-132.6), and 45-64 years (RR 5.3; 3.4-8.3) [24].

Among children under 18 years of age who were diagnosed with HMPV infection, the majority (50%) belonged to the 6-23 months age group. Of the children who were hospitalized, 18% required intensive care unit (ICU) admission and 6% required mechanical ventilation [25]. An outbreak evaluation of HMPV in an alcohol rehabilitation center revealed a 73% (29/40) infection rate. Cough, worsening of dyspnea, and rhinitis with or without fever were evident in 13.79% (4/29) of patients. Ten (34.48%) patients required hospitalization, and one (3.44%) required treatment in the ICU [26]. In a study among 128 critically ill adult patients with a mean age of 55 years, it was observed that 48% of the patients admitted to the ICU had evidence of acute respiratory distress syndrome (ARDS). This study also revealed that 31% of patients required ICU admission, and most of these patients had comorbidities and immunosuppression [27].

Persistent asymptomatic HMPV infections in hematopoietic stem cell transplant (HSCT) patients were noticed. In a study that assessed 768 HSCT patients, HMPV positivity rate was 2.5% (19). Of the infected patients, 72.2% presented with symptoms of URTI like fever, cough, and headache. Five (27.8%) patients developed pneumonia, confirming LRTI [28]. Children with asthma suffered from hypoxic respiratory distress following HMPV infection compared to those who were infected with RSV. Additionally, HMPV infection exacerbates asthma in children and adults [29, 30]. HMPV infection may result in increased mortality rates in patients with pre-existing complex chronic conditions. The risk was found to be similar in both HMPV- and RSV-infected people. However, infection with HMPV predisposes to longer hospital stays and higher financial burden [31]. 

A previous study assessed the duration of viral shedding among the infected population who had malignancy. This study utilized culture and polymerase chain reaction (PCR) to detect viral shedding. HMPV infection revealed viral shedding for 10.5 median days (5-29 days) versus 14 days (5-42 days) with an insignificant (p=0.2) result by culture and PCR, respectively [32]. Nosocomial transmission resulting in the outbreak of HMPV infection among hematologic malignancy patients was reported in Germany. This outbreak was associated with HMPV genotype A2a. Among the infected patients, 65% developed dyspnea and 33% required mechanical ventilation. Four patients died due to pneumonia and associated complications during this outbreak, demonstrating high mortality rates (26.6%) [33].

Immunological responses to HMPV infection

Infections with HMPV and other respiratory viruses stimulate innate immune responses wherein the viral antigens, also known as pathogen-associated molecular patterns (PAMPs) are recognized by the PRRs of the host's immune system. It was identified that during viral infections, the TLRs, including TLRs-3, 4, and 7-9, actively participate in immune responses. TLR3 and TLR4 are known to play a key role in immune responses against RSV and HMPV infections, suggesting variable immunological responses to these infections. In HMPV infection, the TLR4 activation results in the induction of nuclear factor kappa-light-chain-enhancer of activated B cells (NF-κB), and activation of inflammatory chemokines, cytokines, and IFNs, especially in the respiratory epithelium. Downregulation of signaling pathways that activate innate and adaptive immune responses influences the immunity to HMPV infections, further predisposing people to recurrent infections and reinfections [34]. Studies have shown that despite mounting a normal immune response to initial infection with HMPV, failure to stimulate memory cells predisposes people to reinfections [8]. Immunological responses to HMPV infections are poor with respect to innate immunity, which in turn affects the adaptive responses. This is evident by the low secretion of cytokines and anergic or weak stimulation of T cell responses [35].

Laboratory and experimental studies have demonstrated an increase in the alveolar macrophages and raised neutrophils with unaltered NK cells following infection with HMPV. Decreased CD4+T cell subsets and increased T regulatory (Treg) cells were significant changes during adaptive immune responses, limiting protection against potential invasive infection by HMPV [8]. Previous studies have observed that the infection with HMPV fails to evoke adequate immune responses. Like in the RSV infection, HMPV does not result in the production of cytokines, thereby affecting CD4+ T cell responses. This scenario has not been completely understood since HMPV does not possess NS proteins, which play a key role in suppressing the pathways that initiate CD4+ T cells and cytokines. Furthermore, it was identified that the G and SH proteins function to modulate immune responses during HMPV infection [35]. Experiments in vitro in mouse airway epithelial cells and in vivo in mice have revealed that the type III IFN (IFN-λ) is upregulated during HMPV infection as a part of potent antiviral immunologic responses. Despite its proinflammatory nature, IFN-λ contributes to lowering the accumulation of lung macrophages and reducing the loss of respiratory epithelial cells during HMPV infection. Therefore, IFN-λ is projected as an immunomodulatory molecule that has the potential to be used as a therapeutic agent against HMPV and other respiratory viral pathogens [36].

Clinical features

Patients with an acute HMPV infection start to exhibit clinical symptoms three to six days after exposure. HMPV infections have symptoms that are comparable to those of other RTIs. HMPV-associated URTIs can be asymptomatic, but they can also present with fever, headache, sore throat, and body aches and pains. Other symptoms, such as shortness of breath and nasal congestion, may also appear in patients infected with HMPV [37, 38]. HMPV majorly affects the lower respiratory tract, unlike other seasonal viral RTIs. HMPV-induced LRTI presents as fever/feverishness, cough, dyspnea/difficulty in breathing, rhinorrhea, and abdominal pain. More than half of the infected patients may require oxygen supplementation. Interestingly, HMPV pneumonia was comparatively less serious than the pneumonia caused by RSV [20, 21]. HMPV pneumonia in children and adults may show symptoms like cough, rhinorrhea/running nose, dyspnea, retractions, wheezing, and leukocytosis [20]. Differential diagnosis solely based on clinical presentation is impractical for physicians treating patients. Therefore, confirmation of etiologies through appropriate laboratory methods is important to effectively manage patients. 

Laboratory diagnosis

A clinical evaluation, which includes evaluating the patient's symptoms and medical history, is typically required to suspect an HMPV infection. Nasal secretions, nasal and nasopharyngeal swabs may be suitable specimens to diagnose URTIs. Bronchoalveolar lavage, endotracheal aspirates, and sputum may be more suitable for diagnosing LRTIs. The gold standard for diagnosing viral infections is through virus isolation from clinical samples. Laboratory testing, such as polymerase chain reaction (PCR), enzyme-linked immunosorbent assay (ELISA) and rapid antigen tests like immunochromatographic test (ICT), among others, are essential to confirm the diagnosis. Multiplexed nucleic acid amplification test (NAAT) is routinely used to diagnose patients with RTIs [39]. Liquid chromatography-tandem mass spectrometry (LC-MS), a targeted proteomic assay, was found to be effective in the identification and diagnosis of HMPV in nasopharyngeal swabs. This method was suggested as an alternative to nucleic acid-based sequencing [40]. The utility of the direct immunofluorescence (DIF) assay compared to the quantitative reverse transcription-polymerase chain reaction (qRT-PCR) assay was assessed in the diagnosis of HMPV. DIF was able to detect HMPV in 36 (8%) samples compared to 94 (21%) by qRT-PCR. The sensitivity and specificity of the DIF assay were found to be 38% and 99%, respectively [41]. Cross-reacting antibodies to HMPV were demonstrated among children diagnosed with RSV, suggesting antigenic similarities in tune with identical clinical presentation, including acute febrile respiratory illness [42].

Despite the availability and extensive use of molecular methods, including antigen, antibody detection tests, and nucleic acid assays like PCR, they have been documented to be prone to false-positive and false-negative results. A major cause of false-positive reactions is sample collection and handling-related contamination. The antigen and antibody detection-based tests may provide false-positive results due to cross-reacting antigens or antibodies, prior vaccination, and the use of nasal sprays. Inhibitory substances in blood and the haematocrit concentration can also result in false-positive results. PCR-based false-positive reactions are attributed to sample cross-contamination, technical glitches like temperature, primers, probes, and others like variable cycle threshold (Ct) values, and inactive or residual detection. The false-negative results occurring in antigen and antibody detection assays are due to technical problems related to inappropriate sample and reagent storage temperatures, wrong sampling time, and exogenous and endogenous inhibitory factors like nasal sprays and blood haematocrit concentration. PCR-based false-negative results are attributed to technical mistakes while processing the samples, like temperature, primers, probes, and others, and variable Ct values. For cases with low viral loads, such as in the early asymptomatic stages of the infectious disease, advanced molecular techniques like clustered regularly interspaced short palindromic repeats (CRISPR)/CRISPR-associated protein 9 (Cas9)/CRISPR-Cas12a/13a may be more effective than the PCR limit of detection (LoD) score. These techniques may be available worldwide for the detection of minute viral loads in actual samples, such as plasma, serum, urine, or swabs. In the near future, a number of additional genome sequencing techniques could emerge, completely changing the molecular diagnostics of infectious viral illnesses [43, 44].

Epidemiology

Following its discovery in 2001, HMPV infections have been reported from different regions of the world. A vast majority of infections have been noticed in children under five years of age. HMPV infections were also reported in young children, adolescents, young adults, and older populations. HMPV was reported as a cause for RTI alongside other etiologies, including RSV, IAV, IBV, and other respiratory viruses like rhinovirus, HPIV types, adenovirus, and coronaviruses. Interestingly, studies have also observed co-infections of HMPV with other viruses, especially RSV. Evidence of antibodies against HMPV was available among children of different age groups, with a majority of adults revealing antibodies. Seroprevalence was reported from South Africa, China, Japan, and Canada. Data on the surveillance of HMPV genotypes revealed that genotype A was the most predominant genotype, with subtypes A2, A2b, A2c, and A3 (China) being more frequently observed. HMPV genotype B was also prevalent in some parts of the world, including India and Australia. HMPV genotype B subtypes, including B1 and B2, were found in some geographical regions. Reports on genotypes A and B co-circulating in a particular geographical region have also been available. Genotypes A2 and B were found to be co-circulating in Korea, Sweden, Jordan, Ireland, China, Kuwait, Norway, and Italy. Replacement of genotype was reported in Croatia, wherein A2b was replaced with A2c. (Table 1)

**Table 1:** Infection rates, seroprevalence and molecular epidemiology of human metapneumovirus and other respiratory viruses throughout the world This table has been created by the authors. NA: not available; HMPV: human metapneumovirus; RSV: respiratory syncytial virus; IAV: influenza A virus; IBV: influenza B virus; HAdV: human adeno virus; SARI: severe acute respiratory infection; PIVs: parainfluenza viruses; HRV: human rhinovirus; ARTI: acute respiratory tract infection; EBV: Epstein-Barr virus; LRTI: lower respiratory tract infection; RTI: respiratory tract infection; IgG: immunoglobulin G; ICV: influenza C virus; HSV: herpes simplex virus; RT-PCR: reverse transcription polymerase chain reaction; PYO: person-years of observation; IQR: interquartile range

Country and year	Patient characteristics	HMPV	RSV	IAV	IBV	Other respiratory viruses	Seropositivity and molecular epidemiology	Reference
Brazil, 2003	111 children	17% (19/111)	48% (53/111); 7% (8/111) had RSV/HMPV co-infections	NA	NA	NA	NA	Cuevas et al. [45]
USA, 2003	984 illnesses, young children, and old adults	4.5% (44/984) symptomatic; 4.1% (9/217) asymptomatic	NA	NA	NA	NA	NA	Falsey et al. [46]
South Africa, 2004	137 children with RTI	5.8% (8/137) 2-43 months	15.3% (21/137)	13.1% (18/137)	14.5%(20/137)	NA	IgG seen in 92% (24-36 months aged children)	IJpma et al. [47]
Argentina, 2004	100 samples from children aged <5 years	11% (11/100); 91% (10/11) hospitalized	NA	NA	NA	NA	NA	Galiano et al. [48]
Denmark, 2004	374 children with ARTI	2.9% (11/374)	50.80% (190/374), 2 co-infected with RSV and HMPV	NA	NA	NA	NA	von Linstow et al. [49]
Japan, 2004	100 children aged one month to 5 years	NA	NA	NA	NA	NA	HMPV and RSV antibodies in children over 4 months (43% vs. 60%, P < 0.025); 4 months to 1 year (11% vs. 48%, P = 0.006)	Ebihara et al. [50]
Norway, 2004	236 children with RTI	21% (50/236)	15% (36/236)				Outbreak	Døllner et al. [51]
USA, 2004	Children aged <18 years	6.2%; majority in 3-24 months	NA	NA	NA	NA	NA	McAdam et al. [52]
Italy, 2005	1505 children	2.8% (42/1505)	9.5% (143/1505), 2.3% (1/42) HMPV and RSV co-infection	15.3% (230/1505) Influenza viruses 14.2%(6/42) HMPV and influenza virus coinfections	NA	NA	NA	Bosis et al. [53]
Thailand, 2006	220 infants and young children aged 22±11 months	5% (12/220)	NA	NA	NA	NA	NA	Samransamruajkit et al. [54]
USA, 2006	146 adults	3.4% (5/146)	11.6% (17/146)	NA	NA	NA	NA	Falsey et al. [55]
Australia, 2006	727 Australian HMPV strains isolated from 10,319 specimens	NA	NA	NA	NA	NA	Subtype B1 was the most common lineage	Mackay et al. [56]
France, 2007	931 children aged <3 years	6% (55/931);	28.5% (265/931)	NA	NA	18.3% (170/931) HRV	NA	Manoha et al. [57]
Romania, 2007	28 children (9 months to 6 years) negative for Influenza and RSV	25% (7/28)	NA	NA	NA	NA	NA	Tecu et al. [58]
South Africa, 2007	2715 nasopharyngeal samples from HIV infected children	11.3% (306/2715)	21.1% (572/2715)	NA	NA	NA	NA	Madhi et al. [59]
Canada, 2007	All age groups	NA	NA	NA	NA	NA	3.5% (13/96) in 0-5 years, 26.1% (25/96) in 6-10 years, 32.3% (31/96) in 11-15 years, 99.0% (95/96) in 16-30 years, 91.7% (88/96) in 31-60 years, and 93.8% (90/96) in 61+ years	Liu et al. [60]
China, 2007	3330 samples from children	3.3% (110/3330); 46.4% (51/110) positives in <1 year age	1.8% (2/3330) coinfections with RSV	NA	2.7% (3/3330) coinfections with IBV	0.9% (1/3330) coinfection with PIV type 3	VA	Zhu et al. [61]
China, 2008	325 children aged <6 years	NA	NA	NA	NA	NA	74.5% (242/325) anti-HMPV IgG antibody in 0-5 months age; 64% (208/325) in 6-11 months age; 72.7% (236/325 in 12-23 months age; 87.1% (283/325) in 24-35 months age; 90.3% (293/325) in 3-6 years age	Zhang et al. [62]
Italy, 2008	Children <5 years hospitalized for ARTI	25.8% in winter and spring, and 4.7% in the other seasons	48.7% (19/39) HMPV and RSV co-infections	NA	NA	NA	60.4% of the HMPV detected were A2a, 22.9% were A2b, 4.2% were B1, and 12.5% were B2; A1 strains were not detected	Caracciolo et al. [63]
Japan, 2008	379 children with a mean age of 3.5 years (range, 2 months to 9 years) with LRTI	25.8% (98/379)	24.7% (69/379)	NA	NA	4.7% (18/379) HAdV, 3.1% (12/379) enterovirus, 2.1% (8/379) PIVs, 0.7% (3/379) HRV, 0.5% (2/379) ICV, 0.2% (1/379) HSV	NA	Hara et al. [64]
Korea, 2008	1214 children aged <16 years	8.4% (102/1214)	NA	NA	NA	NA	85.29% (87) genotype A2; 14.70% (15) genotype B	Chung et al. [65]
Spain, 2008	99, children aged <12 months	25% (25/99)	35% (35/99)	NA	NA	19% (19/99)	NA	Camps et al. [66]
Sweden, 2008	4989 children aged <3 years	2.9% (143/4,989); 50.3% (72/143) were aged <3 years					95.6% (87/91) genotype A; 4.3% (4/91) genotype B	Rafiefard et al. [67]
Austria, 2008	1612 children aged <2 years	6.8% (109/1612)	NA	NA	NA	NA	NA	Aberle et al. [68]
Ireland, 2008	625 children assessed with immunofluorescence and RT-PCR	4.8% (30/625)	NA	NA	NA	NA	HMPV subtypes A2b and B2; no HMPV subgroups A1 or B1	Carr et al. [69]
Chile, 2009	All ages and children	NA	NA	NA	NA	NA	A3 sublineage (59%)	Escobar et al. [70]
Brazil, 2009	927 children aged <5 years	12.3% (114/927)	NA	NA	NA	NA	NA	Carneiro et al. [71]
UK, 2009	7091 samples from 4282 children	2% (142/7091)	NA	NA	NA	NA	70% (100/142) genotype A	Gaunt et al. [72]
China, 2010	622 children aged <5 years	3.86% (24/622)	NA	NA	NA	NA	A2 genotype HMPV	Shen et al. [73]
France, 2010	374 children aged <2 years	6% (23/374)	47% (177/374)	NA	NA	47% (175/374)	NA	Rigal et al. [74]
Taiwan, 2010	1120 serum samples from age 0-99 years	NA	NA	NA	NA	NA	Overall seropositive 68.1%; 53.2% of preschool children were seronegative; 88.3% of school-aged individuals and 93.7% of adults had antibodies to HMPV	Huang et al. [75]
Jordan, 2012	220 children ≤13 years	12.7% (28/220)	NA	NA	NA	NA	HMPV genotype A in 26 (93%) and type B in 8 (28.6%)	Qaisy et al. [76]
Türkiye, 2012	100 children aged 0-10 years	11% (11/110) by cell culture; 2% by RT-PCR	NA	NA	NA	NA	NA	Aksoy Gökmen et al. [77]
India, 2014	440 children <12 years with SARI	3.63% (16/440) with 5.1% in <5 years and 5.08% in 6-12 years	14.3% (63/440) with 24.68% in <5 years	NA	NA	NA	Genotype B (68.8%)	Jain et al. [78]
China, 2014	151 children	4% (6/151)	66 (43.7%)	13 (8.6%)	1 (0.7%)	2 (1.3%) with PIV type 3, 1 (0.7%) with PIV type I, 1 (0.7%) with HAdV	NA	Wang et al. [79]
Canada, 2015	7575 specimens from 2292 respiratory outbreaks involving adults	436 of 195 outbreaks(8.5%) with mean age of 80.7 years	NA	NA	NA	NA	Genotype A2b was the most prevalent, 28 (54.9%); genotype A2b2 was also identified	Neemuchwala et al. [80]
Kenya, 2015	Children <5 years with SARI	0.9/PYO	2.8 cases/100 PYO	1.0 cases/100 PYO	NA	1.1 case/100 PYO for PIV, 1.5 cases/100 PYO for	NA	Breiman et al. [81]
China, 2015	2613 nasopharyngeal aspirates from hospitalized children <14 years with LRTI	5.2% (135/2613)	NA	NA	NA	NA	Genotype A 72.6% (98/135), genotype B 27.4% (37/135), trend over 3.5 years A2b to A2b or B1 and then to predominantly B1; A2b and B1 were found to co-circulate	Zeng et al. [82]
Jordan, 2015	3168 children <2 years admitted with fever and/or acute respiratory illness	8.6% (273/3168)	NA	NA	NA	NA	Genotypes A2, B1 and B2, but not A1, were detected during the 3-year period; subgroup A and age <6 months required supplemental oxygen	Schuster et al. [83]
Kuwait, 2015	URTIs and LRTIs in all age groups. Children, the elderly, and immunocompromised individuals	5%	NA	NA	NA	NA	62% of HMPV sequences belonged to the A genotype and 38% to the B genotype; A2b and B2 subtypes were detected and circulated during the study period, whereas A1 and B1 subtypes were not detected	Al-Turab et al. [84]
Bulgaria, 2015	416 nasopharyngeal swabs of children aged <4 years presenting with ARTI	9.8% (28/287)	19.2% (55/287)	H1N1: 14.7% (61/416), H3N2: 3.4% (14/416)	11.8% (49/416)	HPIV1 5.9% (17/287), HPIV2 1.7% (5/287), and HPIV3 4.9% (14/287)	NA	Korsun et al. [85]
Japan, 2015	289 hospitalized children <5 years with LRTI	16.6% (48/289)	NA	NA	NA	10.0% (29/289) ICV	NA	Shimizu et al. [86]
Germany, 2016	272 children and adolescents (0-18 years) with ARTI or influenza-like illness	15%, (41/272)	32%, (87/272); 1.47%, 4/272 coinfection of RSV and HMPV	11% (30/272)	NA	HRV (18%; 49/272); human bocavirus (7%, 20/272), and HAdV (7%, 19/272	NA	Vogel et al. [87]
USA, 2016	571 preschool children aged ≤5 years with severe respiratory illness	11% (63/571); 37% had severe prematurity (<32 weeks of gestation)	NA	NA	NA	NA	NA	Pancham et al. [88]
Saudi Arabia, 2016	174 nasopharyngeal aspirates from hospitalized children	10.9% (19/174) in children aged 7-24 months	22.4% (39/174) in infants less than 6 months old	19.5% (34/174) in children aged 25-60 months	NA	Coronaviruses 8% (14/174), PIVs 6.3% (11/174) in infants less than 6 months old	NA	Amer et al. [89]
Morocco, 2016	683 children aged 2-59 months	8·9% (61/683)	18·2% (124/683)	NA	NA	NA	NA	Jroundi et al. [90]
UK, 2016	127 children aged 4.01±5.05 years	3.9% (5/127)	46.4% (59/127)	NA	NA	NA	NA	Othman et al. [91]
Norway, 2017	1816 children <16 years with LRTI and hospitalization	9.4% (171/1816), 6.24% had coinfection	47.3% (859/1816), 0.6% (11/1816) had both HMPV and RSV	NA	NA	NA	Genotype A 67 (46%), 12 (17.91%) A2a, 28 (41.79%) A2b, and 27 (40.29%) A2 (unassigned); 54% (43/80) genotype B-26 (32.5%) were B1, and 54 (67.5%) were B2	Moe et al. [92]
Croatia, 2019	Children under 5 years of age	NA	NA	NA	NA	NA	HMPV genotype A subtype A2c; A2b replaced with A2c	Jagusic et al. [93]
Saudi Arabia, 2019	91, children aged 9 months to 16 years; 77.8% hospitalized	9.9% (9/91)	NA	NA	NA	NA	NA	Alsuheel et al. [94]
USA, 2020	2358 children (0-17 years) and 2320 adults (>18 years) with pneumonia	298/2358 (12.6%) children with a median age of 23 months; 88/2320 (3.8%) adults with a median age of 56.5 years	NA	NA	NA	Coinfection (97/125; 78%), including RSV, HAdV, and/or HRV; bacterial pathogens (13/125; 10% or both bacterial and viral pathogens (8/125; 6%)	NA	Howard et al. [20]
Italy, 2023	11,008 patients with a median age of 9.3 (0.06–97.4) years	2% (222/11,008); 0-6 months: 49 (22.1%), 6m-3 years: 46 (20.7%), >16 years: 105 (47.2%)	11.2% (1300/11,577); 0-6 months: 481 (37.4%), 6m-3 years: 278 (21.6%), >16 years: 428 (32.9%)	NA	NA	NA	36 (65.5%) were HMPV-A/subgroup A2c strains, 19 (34.5%) were HMPV-B/subgroup B2 and 1 B1	Pierangeli et al. [95]
China, 2024	96 pediatric patients with HMPV infection with a median age of 33.5 months (IQR: 12–48 months)	87.5% of infected children were under 5 years old	NA	NA	NA	Coinfection with EBV in 43 (15.6%)and HRV type A (12.5%); Bacterial coinfection in 74 (77%), with *Haemophilus influenzae* (52.1%) and *Streptococcus pneumoniae* (41.7%)	NA	Ji et al. [96]
China, 2024	155,165 hospitalized children 0–17 years of age with ARTI	1.62% (2524/166165); median age 27 months; 15.06% (380/2,524) coinfections; *Mycoplasma pneumoniae* (16.58%, 63/380), HRV (13.95%, 53/380), and HAdV (12.63%, 48/380) coinfections; 41 (10.79%) with two or more pathogens	9.38% (14,564/155165); median age 8 months; 9.63% (1,402/14,564) coinfections; HAdV (19.04%, 267/1,402), *M. pneumoniae* (16.98%, 238/1,402), and Enterovirus (11.13%, 156/1,402); 8.92% (125/1,402) two or more pathogens	NA	NA	NA	NA	Kuang et al. [97]

Prevention of HMPV

HMPV prevention methods are vital to mitigate the spread of the disease, especially in high-risk environments such as schools, hospitals, elderly care facilities, clinics, daycares, and so on. Since the underlying reason for the virus spread is still unknown, it is important to follow practices and health measures to ensure low exposure and safety from it. Public interventions are the first step to preventing the spread through the simple etiquette of washing hands thoroughly and wearing masks in “poorly ventilated spaces” and areas of risk - hospitals, medical offices, and clinics. Improving ventilation to increase airflow, disinfecting regularly, and avoiding contact with the face (eyes, nose, mouth) that is not clean are the best measures to prevent the infection [37]. However, children are at the highest risk [98]. Even with simple measures that can prevent exposure, it is still hard to control the actions of children and make sure they “do not touch their face” or “wash their hands properly.” Researchers are still in pursuit of ascertaining the outcome, and the emergence of HMPV methods to help children prevent the virus must become a priority. Regardless, the Centers for Disease Control and Prevention (CDC), Atlanta, emphasizes supportive treatment and appropriate respiratory precautions to prevent transmission of the disease while also highlighting the importance of monitoring high-risk individuals such as children and the elderly. Along with the WHO, who similarly focus on supportive care in low-resource settings where there is a lack of diagnostics and treatment availability. WHO recommends monitoring cases of respiratory disease and additional interventions [99, 100].

There are also diagnostic challenges associated with the post-COVID-19 season. There have been fewer and fewer research studies, statistics, and knowledge on HMPV, especially during and after the pandemic. It is hard to keep track of the likelihood of people who get tested for HMPV, or if other tests are prioritized above others. For instance, RT-PCR holds a “gold standard” for diagnosing HMPV as it is the most reliable and accurate test. RT-PCR uses reverse transcription to create a deoxyribonucleic acid (DNA) template from an RNA sample [37, 101]. This test can detect viral RNA with high accuracy via respiratory specimens such as nasal swabs, throat swabs, and sputum [11]. RT-PCR was also considered the “gold standard” for COVID-19 testing too for the detection of the genetic material from the SARS-CoV-2 virus. However, the differences in use lie in the scale of magnitude, severity of the disease, and availability. COVID-19 was a global pandemic compared to HMPV, which is still much less well-known. COVID-19, for these reasons, saw large-scale testing even in asymptomatic individuals in all environments. It was prioritized by the majority of healthcare providers, with the tests being integrated into routine testing. Moreover, although HMPV and COVID-19 are both respiratory illnesses, HMPV does not have the same severe global health impact as COVID-19. HMPV is usually employed only for specific, targeted cases, whereas COVID-19 saw people getting tested even out of caution. The availability of testing was more widespread during the time of COVID-19 and included free and low-cost options in many countries. However, HMPV testing is more specialized and less available to the general public. In addition, the changes in surveillance infrastructure are also noteworthy. After the lockdown measures of the pandemic were lifted, the spread of HMPV increased [102]. The public health monitoring and surveillance infrastructure that was used for COVID-19 would greatly contribute to the spread of HMPV, as certain trends can be identified and monitored. Infrastructure such as influenza and viral respiratory disease surveillance, syndromic surveillance, lab reporting, health care systems reporting, research platforms, vital statistics, and new surveillance systems designed to answer specific questions can provide more insights into the disease and maintain its spread [99]. Enhanced surveillance will prevent overburdening healthcare systems, enabling them to devote for research into the disease while providing care for infected/symptomatic patients [103].

Vaccination for HMPV

Although there is currently no vaccine approved against HMPV, some studies are being carried out to develop an effective vaccine. A formalin-inactivated HMPV vaccine was found to induce severe lung inflammation due to Th2 responses in lab animals. Development of nano-emulsion inactivated HMPV vaccine, which could be immunogenic and elicit protective immune responses, was suggested. HMPV F protein was used to develop a subunit vaccine that was able to elicit strong humoral immune responses in experimental animals. However, vaccination with HMPV G protein did not elicit adequate neutralizing antibodies (NAbs). Animal experiments with HMPV virus-like particles (VLPs) expressing M and F proteins in suspension-adapted human embryonic kidney epithelial (293-F) cells were sufficient to protect against lung infection in mice. Immunization with VLPs exhibiting F and M proteins elicited both humoral and cell-mediated (CD4+ T cells) immune responses. A recombinant HMPV vaccine prepared with P protein and using bacillus Calmette-Guerin (BCG) as a carrier was found to be efficient in eliciting CD4+ and CD8+ T cells in animals. Some studies have noticed that the HMPV G protein was unsuitable due to low antigenicity, as noticed by insufficient production of NAbs. However, recombinant vaccines utilizing the G protein were noted to be efficient in developing protective immune responses. A few live attenuated recombinant vaccines against HMPV have also been studied. The non-recombinant live attenuated viral strains obtained through mutation following serial viral cultures were promising, but the reversal to wild-type virus remains the major drawback. Animal experiments using recombinant HMPV vaccine candidates lacking G (rhMPV-ΔG), G and SH (rhMPV-ΔG/SH), and M2-2 (rhMPV-ΔM2-2) proteins demonstrated their efficacy in restricting the viral replication in the respiratory epithelial cells. A recombinant HMPV vaccine utilizing the avian P protein (rhMPV-Pavian) was authorized for human trials, but the results revealed that this virus was unable to infect humans. The available studies reveal the complexity associated with the development of a successful vaccine against HMPV [104]. Laboratory-based animal experiments using mouse cells infected with VLPs of HMPV vaccine studies indicate lung CD8+ T cell impairment mediated by programmed death 1 (PD-1) during reinfections despite initial protection [105].

Treatment for HMPV infections

Like most other respiratory viral diseases, HMPV generally causes self-limiting infections. There is neither an approved human vaccine nor any specific antiviral therapeutic agent to treat HMPV infections. Patient management involves symptomatic treatment using anti-pyretic and analgesic drugs, including N-acetyl-para-aminophenol (APAP) or paracetamol and ibuprofen. Treatment also involves the use of decongestants (pseudoephedrine or phenylephrine), including nasal sprays, mucolytic agents (acetylcysteine, dornase alfa), expectorants (guaifenesin), and cough depressants (dextromethorphan). Antihistamines, including diphenhydramine, benadryl, loratadine, cetirizine, fexofenadine, which are commonly used to treat allergies, although not very successful at treating viral infections, are used in patient management [106]. Children and adults with co-morbid conditions like asthma, COPD, HIV infection, and organ transplant patients can be predisposed to complications of HMPV infection. Close monitoring of such patients is extremely important, considering that these individuals may require hospitalization and intensive care treatment with oxygen supplementation. The worldwide health care system is heavily burdened by the lack of a vaccine or antiviral medication to prevent HMPV infection. Due to its lower research and development costs compared to traditional drug discovery (which is associated with huge costs and takes five to 10 years to develop), drug repurposing has grown in popularity as a means of treating endemic and emerging diseases. Direct-acting repurposed antiviral (DARA) and host-targeting repurposed antiviral (HTRA) are the two main kinds of repurposed antiviral targets [107]. Eleven candidates were shown to have dose-dependent inhibitory efficacy against HMPV infection. Five anti-HMPV candidates with low in vitro cytotoxicity had their mechanisms of action clarified. The anti-HMPV activities of three post-entry inhibitors (mycophenolic acid, mycophenolate mofetil, and 2,3,4-trihydroxybenzaldehyde) and two entry inhibitors (Evans Blue and Aurin tricarboxylic acid) were assessed. According to the study, mycophenolic acid and mycophenolate mofetil offer significant potential for therapeutic repurposing because of their substantial ant-HMPV actions, and inhibitory levels were obtained below the authorized oral dosage for people [108].

The FDA-approved antiviral medications and control compounds were the focus of the advanced computational techniques used to identify potential antiviral therapies targeting HMPV. These techniques included virtual screening, molecular docking, molecular dynamics (MD) simulations, density functional theory (DFT) analysis, and absorption, distribution, metabolism, and excretion (ADMET) profiling. Remdesivir and peramivir, along with oseltamivir, zanamivir, baloxavir, ritonavir, molnupiravir, sofosbuvir, lamivudine, tenofovir, entecavir, acyclovir, valacyclovir, and famciclovir were analyzed. Remdesivir and peramivir were shown to be the most promising options for treating HMPV infections. In the past, both of these medications have demonstrated broad-spectrum antiviral efficacy against RNA viruses [109]. The effectiveness of the host-targeting antiviral medication probenecid in preventing (pre-virus) or treating (post-virus) HMPV replication was evaluated. According to the study, probenecid at concentrations of ≥0.5 μM significantly inhibited HMPV replication in vitro in LLC-MK2 (a continuous epithelial cell line derived from the renal tissue of adult rhesus monkeys (*Macaca mulatta*) cells), and probenecid treatment or prophylaxis at doses of 2-200 mg/kg decreased HMPV replication in an albino, laboratory-bred (BALB/c) mice [110].

Future directions

The molecular identification of several previously unidentified or unknown infectious agents, as well as known agents that have spread to new populations or geographic areas, has been accomplished thus far. However, a thorough diagnosis of respiratory infections necessitates a patient's medical history, physical examination, radiologic results, and laboratory data, the latter of which essentially includes the pathogen's molecular identity. Pathogenic agents and emerging infectious diseases, including HPAI and HMPV, necessitate prompt and accurate techniques to handle cases with additional acute care strategies and obtain exact molecular identification. To reduce delays and waiting time for laboratory responses to severe or critical cases, laboratories should be correctly equipped with very sensitive, quick diagnostic devices that enable front-line clinicians to make a quick bedside diagnosis. Therefore, the first ideal would surely be for scientific groups to collaborate to develop fast, highly specific, sensitive, and adaptable diagnostic technologies that are suitable for front-line use, especially in severe or critical cases during a pandemic-like situation.

Rapid diagnostic tests (RDTs) with improved sensitivity, accuracy, and ease of use are desperately needed. RDTs should be readily available in emergency rooms and other elder care institutions to avoid delaying acute care in urgent situations while awaiting test results. Furthermore, PCRs or lateral flow immunoassays (LFIAs) might be developed to differentiate between pathogens using just one sample. The current advancements in genome sequencing techniques like whole-genome sequencing (WGS) or next-generation sequencing (NGS) could completely revolutionize the molecular diagnostics of infectious viral illnesses. Furthermore, standard traditional methods used in laboratories may be modified to enable faster and more sensitive diagnosis, even outside of the lab. Accurate molecular diagnosis is essential, especially for front-line physicians, as emerging infectious illnesses may increase shortly [43]. Several significant problems with molecular diagnostics were exposed by the COVID-19 pandemic, primarily related to nomenclature, role, quantity, and quality when diagnosing newly emerging infectious diseases. However, COVID-19 gave the scientific community several molecular diagnostics lessons. Public health will be very different in the future than it was before the pandemic. Rapid molecular diagnostics, even to a significant extent, referring to self-diagnosis goals, was the most important public health maintenance policy that held society together throughout the COVID-19 epidemic.

To directly prevent and promptly manage future infectious illnesses, epidemics, and pandemics, new preventive medicine-based strategies need to be investigated and validated. The study of shared antigenic sites between pathogens can be crucial for future vaccines, tests, and medications, and the global immunological blueprint that was presented as a novel strategy for preventing future (re)emerging infectious diseases, epidemics, and pandemics appears to be of high scientific importance. Communities are equipped with these weapons to combat the upcoming pandemic right away [44]. It is urgently necessary to build a plan based on a multifaceted approach to prevent the extraordinary rise in the number of emerging and re-emerging viruses with pandemic potential from surpassing the capabilities of national and international health systems. An improved risk assessment framework, a well-designed global warning system to improve prediction capacities, advanced technologies at human-animal interfaces, and improved public health reporting systems dedicated to the prompt global dissemination of information on emerging hazards are crucial. These systems are crucial for tracking disease trends in the context of human and animal disease outbreaks, particularly in wildlife, domestic, and farm animals, which serve as hotspot regions where human activities encourage the emergence of zoonotic microorganisms and their subsequent spread through cross-species transmission. Expert, technical, and knowledge exchange, as well as the real-time distribution of data via cooperative networks, allow policymakers to make critical decisions about public health both before and during pandemics and other health catastrophes [111].

## Conclusions

The available literature presents clear evidence of human exposure to HMPV before it was discovered as an etiological agent responsible for RTI. Serological studies demonstrated the presence of antibodies against HMPV in a majority of people, confirming their exposure to this virus following birth. HMPV is a common respiratory virus like others, including the RSV, influenza viruses, and rhinovirus. HMPV can infect people of different age groups, with a predilection to cause infections among children under five years of age. The presence of predisposing factors like hematological malignancy, HIV infection, and other immunosuppressive and debilitating conditions like asthma and COPD may result in the severity of HMPV infection. Genomic surveillance indicates co-circulation of two genotypes and their subtypes, with evidence of the presence of single genotypes in some geographical regions. Currently, there is no HMPV vaccine approved for human use. Many studies are in their infancy, with vaccine candidates still in their preclinical phases. The COVID-19 pandemic has influenced the prevalence of HMPV and other respiratory pathogens. This can be confirmed by the availability of studies showing their prevalence before and after the pandemic. However, the data during the pandemic is scarce, probably due to the stringent infection control and preventive measures that were in place during the pandemic. In the majority of reports, HMPV was associated with infection in the pediatric age group, confirming its significance among children. The available literature indicates a significant gap in the epidemiology of HMPV, especially from developing and low-socioeconomic regions of the world. 
